# Concurrent intravenous immunoglobulin and platelet transfusion for refractory alloimmune thrombocytopenia in patients undergoing allogeneic hematopoietic stem cell transplantation

**DOI:** 10.1016/j.htct.2025.103961

**Published:** 2025-08-09

**Authors:** Moazzam Shahzad, Muhammad Kashif Amin, Maggie Nelson, Abhinav Vyas, Joe S. Al-Ramahi, Nausheen Ahmed, Rajat Bansal, Haitham Abdelhakim, Leyla Shune, Al-Ola Abdallah, Anurag K. Singh, Sunil H. Abhyankar, Joseph P. McGuirk, Muhammad Umair Mushtaq

**Affiliations:** a Division of Hematologic Malignancies & Cellular Therapeutics, University of Kansas Medical Center, Kansas City, KS, USA; b H Lee Moffitt Cancer Center, Tampa, FL, USA; c Mikael Rayaan Foundation Global Transplantation and Cellular Therapy Consortium, Kansas City, KS, USA

**Keywords:** Intravenous immunoglobulin, Platelet transfusion, Refractory alloimmune thrombocytopenia, Allogeneic hematopoietic stem cell transplantation

## Abstract

**Background:**

Severe refractory alloimmune thrombocytopenia is a challenging and life-threatening complication in patients with hematologic disorders who are undergoing allogeneic hematopoietic stem cell transplantation. This study aimed to investigate the utility of continuous intravenous immunoglobulin and platelet transfusions as a therapeutic approach for alloimmune thrombocytopenia in patients undergoing allogeneic transplants.

**Methods:**

A single-center retrospective analysis was conducted of ten adult allogeneic transplant patients hospitalized with transfusion-refractory alloimmune thrombocytopenia. Intravenous immunoglobulin (2 g/kg) was administered as a slow continuous infusion over 48 h along with a continuous apheresis platelet infusion (one apheresis unit over eight hours). Clinical response was defined as the resolution of bleeding or patients being able to undergo the required procedure without bleeding complications.

**Results:**

The median time after the transplant was 27.5 (range: 7–299) days. Myeloablative and reduced-intensity conditioning were performed in 5 (50 %) and 5 (50 %) patients, respectively. The median platelet count at the time of infusion was 4.5 × 10^9^/L. All patients were able to achieve clinical response with the median maximum platelet count within ten days of the infusion being 41.0 × 10^9^/L. The median time to best response was three days with a median platelet count of 27.0 × 10⁹/L.

**Conclusions:**

Continuous intravenous immunoglobulin and platelet infusions over 48 h may be able to overcome life-threatening refractory alloimmune thrombocytopenia in transplant patients and may provide a bridging measure until platelet engraftment or for life-threatening hemorrhage or invasive procedures with high bleeding risk.

## Introduction

Allogeneic hematopoietic stem cell transplantation (allo-HSCT) is an established option for treating various malignant and non-malignant hematopoietic conditions [1]. Known complications of allo-HSCT include infections, graft failure or rejection, graft-versus-host disease (GvHD), and cytopenias [1,2]. Immune-mediated cytopenias following allo-HSCT probably result from immune dysregulation needing multimodal treatment approaches. Specifically, severe transplant-mediated immune thrombocytopenia (ITP), also known as alloimmune thrombocytopenia (AITP), presents as a frequent complication following allo-HSCT [3]. ITP is characterized by a persistent platelet count of less than 100 × 10⁹/L and an increased risk of bleeding [4]. ITP is a general term that includes conditions where the underlying etiology may involve autoimmunity, such as in idiopathic ITP, wherein autoantibodies target platelets. However, AITP is caused explicitly by alloimmunization, where the immune system develops antibodies against transfused or transplanted donor platelets, leading to accelerated destruction or inefficient megakaryocyte production [3,5]. Allo-HSCT recipients with AITP often show no response to random donor platelet transfusions and limited response to HLA (human leukocyte antigen)-matched platelets [6]. Platelet refractoriness, a poor response to platelet transfusions resulting in lower post-transfusion count increments, is a concern in immune-mediated thrombocytopenia [7]. So, severe refractory thrombocytopenia due to alloimmunization remains a life-threatening complication of hematologic disorders requiring substantial platelet transfusions.

Management of ITP involves various options, including corticosteroids, splenectomy, platelet transfusions, thrombopoietin receptor agonists such as eltrombopag and romiplostim, or immunomodulatory therapies such as immunosuppressive agents (e.g., Rituximab), intravenous anti-D, and intravenous immunoglobulin (IVIG) [8, 9, 10]. Clinical management, particularly in severe thrombocytopenia cases refractory to platelet transfusion, often overlaps with idiopathic thrombocytopenia and AITP. Intravenous immunoglobulin (IVIG) is used in both contexts to modulate the immune response and temporarily increase platelet counts by mechanisms such as Fc receptor blockade and reduction of platelet destruction [7]. Studies have shown that IVIG can be administered when a more rapid increase in platelet count is necessary, especially during active bleeding or in preparation for an invasive or life-saving procedure [11, 12, 13]. When first-line corticosteroid therapy is insufficient or contraindicated, IVIG combined with concomitant platelet transfusion can effectively increase platelet counts in immune-mediated thrombocytopenia [13]. There are limited reports in the literature regarding the concomitant use of platelet transfusion and IVIG infusion investigated in allo-immunized HSCT patients [14]. In this study, the clinical outcomes of IVIG with concurrent platelet transfusion was assessed as a therapeutic approach for AITP for patients undergoing allo-HSCT.

## Methods

A single-center retrospective analysis was conducted of all adult patients who had undergone allo-HSCT and had transfusion-refractory thrombocytopenia presumed to be secondary to AITP between April 2021 and August 2023. This study was approved by the University of Kansas Medical Center institutional review board, and all patients consented to receive blood products before receiving IVIG. Data were collected through a retrospective review of electronic medical records. Platelet refractoriness was defined as a corrected count increment (CCI) of ≤7500 one-hour after two consecutive platelet transfusions. Inclusion criteria included adult patients greater than 18 years old with presumed AITP and the need for urgent concurrent IVIG and continuous platelet transfusion for active bleeding or the requirement of a procedure. IVIG (2 g/kg) was given as a slow continuous infusion over 48 h with concomitant apheresis platelet infusion (one apheresis unit over eight hours). The platelet antibody screen was performed using Solid Phase Immune Adherence Assay. Platelet levels were checked one hour after the completion of each transfusion. Clinical response was identified as the resolution of bleeding or the ability of patients to undergo their procedure without bleeding complications. Data analysis was conducted using Microsoft Excel and SPSS. This study aimed to evaluate the effectiveness of IVIG with concurrent platelet transfusion as a treatment for AITP in patients undergoing allo-HSCT.

## Results

**Figure 1:**
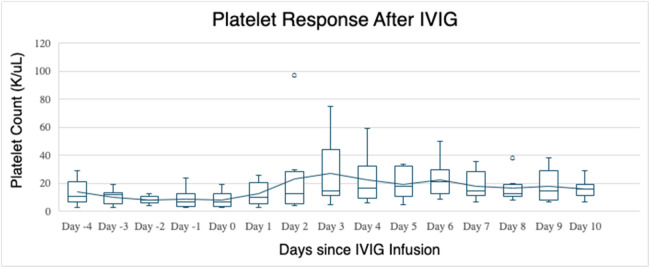
Platelet count after concurrent IVIG infusion.

**Figure 2:**
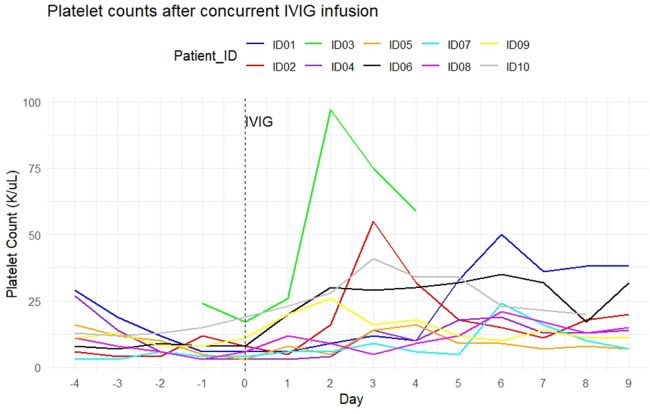
Platelet count after concurrent IVIG infusion: individual patient data.

This study included ten adult patients with transfusion-refractory AITP with a median age of 56 (range: 23.0–66.1) years. Five of the patients (50 %) were males. The median time after the transplant was 27.5 (range: 7–299) days. The primary hematologic malignancies were acute myeloid leukemia (*n* = 3; 30 %), myelodysplastic syndrome (*n* = 6; 60 %), and chronic neutrophilic leukemia (*n* = 1; 10 %). Myeloablative and reduced-intensity conditioning were used in five (50 %) patients each. The indication for an urgent increase in platelets included vaginal bleeding (*n* = 1; 10 %), epistaxis (*n* = 2; 20 %), intravitreal hemorrhage (*n* = 2; 20 %), melena (*n* = 1; 10 %), blood loss anemia of unknown origin (*n* = 1; 10 %), and the need of interventional radiology guided procedures (*n* = 3; 30 %). All patients in this analysis were hospitalized, and seven had sepsis requiring broad-spectrum antibiotics. Eight patients (80 %) had positive antiplatelet antibodies. Reasons for hospitalization included GvHD (*n* = 1; 14 %), neutropenic fever or infection (*n* = 5; 71 %), or active hemorrhage (*n* = 5; 71 %). The median platelet count at IVIG/platelet infusion was 4.5 × 10⁹/L. The baseline characteristics and treatment response with IVIG are shown in Table 1. All patients had resolution of their hemorrhage or were able to achieve a platelet response high enough to receive their procedures without bleeding complications. No adverse events were observed from IVIG infusions in all ten patients. The median time to best response was three days with a median platelet count of 27.0 × 10⁹/L (Figure 1). The individual responses to IVIG infusion are shown in Figure 2.

**Table 1:** Baseline characteristics and treatment response to intravenous immunoglobulin infusion.

Variable	Patient ID									
	ID-01	ID-02	ID-03	ID-04	ID-05	ID-06	ID-07	ID-08	ID-09	ID-10
Age (years)/Sex	28/F	59/M	52/M	64/F	52/F	23/M	47/F	66/M	64/M	61/M
Days between transplant and IVIG	8	11	299	12	7	149	129	17	40	38
Primary diagnosis	AML	MDS	AML	MDS	AML	AA + MDS	MDS	MDS	MDS	CNL
Type of Allogenic transplant	MUD	MSD	MSD	MUD	MSD	MUD	MUD	MUD	Haplo	Haplo
Conditioning type	MAC	MAC	MAC	RIC	MAC	RIC	MAC	RIC	RIC	RIC
Conditioning regimen	Cy/Bu	Cy/Bu	Cy/Bu	Bu/Flu	Cy/Bu	Cy/Flu/TBI	Cy/Bu	Cy/Flu/Mel	Flu/Mel/TBI	Bu/Flu/Cy
Graft source	BM	PSC	PSC	PSC	PSC	BM	BM	PSC	PSC	PSC
Indication of urgent need of platelet increase	Vaginal bleeding	Epistaxis	Ommaya reservoir placement	Trifusion catheter removal	Intravitreal hemorrhage	Intravitreal hemorrhage	Epistaxis	Melena	Blood loss anemia	Lumbar Puncture
Platelet antibody positive?	Yes	Yes	Yes	Yes	Yes	No	Yes	Yes	No	Yes
Platelet count at start of IVIG infusion (x 10^9^/L)	5	3	17	3	3	9	3	4	7	19
Days between IVIG and platelet >10 × 10^9^/L	2	1	0	3	1	1	3	1	1	0
Days between IVIG and platelet 30 × 10^9^/L	5	2	1	13	16	2	NA	6	2	3
Days between IVIG and platelet 50 × 10^9^/L	6	2	2	15	20	112	NA	NA	NA	NA
Maximum platelet count within 10 days	52	55	116	19	16	42	27	40	30	43
Maximum platelet count within 21 days	87	55	116	194	54	42	27	40	30	43
HLA antibodies	Negative	Positive	NA	NA	NA	Negative	Positive	Positive	Negative	Positive

IVIG: Intravenous immunoglobulin infusion; F: Female; M: Male; AML: Acute myeloid leukemia; MDS: Myelodysplastic syndrome; AA: Aplastic anemia; CNL: Chronic neutrophilic leukemia; MUD: Matched unrelated donor; MSD: Matched sibling donor; Haplo: haploidentical transplant; Cy: Cyclophosphamide; Bu: Busulfan; Flu: Fludarabine; TBI: Total body irradiation; BM: Bone marrow; PSC: Peripheral stem cell; IVIG: Intravenous immunoglobulin; HLA: human leukocyte antigen; NA: Not available.

## Discussion

Thrombocytopenia in the context of allo-HSCT is an effect of conditioning regimens and presents a multifaceted challenge with significant clinical implications. Its mechanism is usually multifactorial, underscoring the importance of ongoing research in elucidating and addressing its diverse contributors. Proposed causes of thrombocytopenia include prolonged isolated thrombocytopenia, secondary failure of platelet recovery, and poor graft function [15]. Prolonged isolated thrombocytopenia is characterized by persistently low platelet counts after allo-HSCT (<20 × 10⁹/L) with normal counts in other cell lineages for >90 days. It is seen in approximately 5–20 % of patients who undergo allo-HSCT [15,16]. Its mechanisms remain elusive, potentially involving platelet destruction, ITP-related complications, and/or alterations in the bone marrow microenvironment [17]. Secondary failure of platelet recovery affects approximately 20 % of patients and carries an elevated risk of mortality influenced by factors such as GvHD prophylaxis, donor selection, organ dysfunction, and treatment strategies. Recent advances in transplantation and cytomegalovirus management have altered the incidence and risk factors for secondary failure of platelet recovery [18,19]. GvHD may contribute to reduced platelet production and increased consumption [18]. Poor graft function also presents as persistent cytopenia after allo-HSCT, with a complex etiology encompassing pre-transplant, peri‑transplant, and post-transplant factors that create an inflammatory and immune microenvironment [15,19]. Patients with poor graft function experience poorer survival outcomes influenced by factors like the graft cell dose, ferritin levels, and splenomegaly. These conditions collectively underline the intricate nature of thrombocytopenia in stem cell transplantation, emphasizing the ongoing need for research to enhance patient care and outcomes.

This complexity is further highlighted by the persistence of thrombocytopenia even with routine platelet transfusions. In a comprehensive study involving 50 allo-HSCT patients with and without prolonged thrombocytopenia, 42 patients with idiopathic thrombocytopenic purpura, and 22 healthy individuals, the findings revealed that several factors, including the index for plasma glycocalin normalized for individual platelet count, plasma thrombopoietin levels, and circulating B cells producing anti-GPIIb-IIIa antibodies, were notably higher in patients after allo-HSCT [20]. Furthermore, the study by Leytin et al. in a murine model shows IVIG may increase the platelet count in animals that have antibodies for the GPIIb-IIIa receptor, which suggests that further studies should be carried out to investigate if this is a contributing factor in a human model [21].

While the concurrent use of IVIG and platelet administration was first documented in 1984 for leukemic patients resistant to standard platelet transfusions, Baumann et al. were the first to report on using this combination for ITP [22]. The study included six patients and established the effectiveness of concurrent IVIG and platelet administration. A recent retrospective review of 40 patients revealed that the simultaneous use of IVIG and platelets was safe and effective [13]. The combination therapy was observed to increase platelet count and effectively control bleeding symptoms rapidly. This result offers a promising insight that could aid in developing more effective treatment options for patients suffering from similar conditions. A case series by Ancevski et al. demonstrated that concurrent IVIG infusion and continuous platelet transfusion temporarily increased platelet counts in patients with AITP and bleeding complications [14]. Their findings closely align with our study, where a similar treatment stabilized platelet counts in refractory AITP patients. While earlier reports focused on using this approach in ITP, the study by Ancevski et al. provides direct insight into AITP management, highlighting its effectiveness in modulating immune responses and controlling bleeding. This supports the relevance of using IVIG and platelet transfusions in post-transplant patients with transfusion-refractory thrombocytopenia.

The few studies that researched the concurrent use of IVIG and platelet transfusion have variations in their definitions of a platelet response, patient populations, and comorbidities. These reports studied the use in ITP in adult and pediatric populations [13,22, 23, 24]. Bierling et al. reported platelet counts of >50 × 10⁹/L within a few days [23]. The findings of Spahr et al. demonstrate that average platelets increased to 55 × 10⁹/L after 24 h and peaked at 69 × 10⁹/L 48 h after treatment [13]. Most studies did not report clinical outcomes, such as controlling life-threatening bleeding or undergoing a procedure without bleeding complications. However, the study of Spahr et al. reported that all patients could achieve this clinical response in an ITP setting; the present study did not demonstrate such a high platelet count, likely due to a more significant challenge to achieve a response in the setting of AITP. Additionally, these studies do not report whether there is resolution of their life-threatening bleeding.

Of note, two patients in the current study (ID-06 and ID-08) did not demonstrate positive anti-platelet test results. However, they were still managed as refractory AITP as immune reconstitution following allo-HSCT is often delayed, and antibody testing is unreliable due to B-cell aplasia causing impaired antibody production [2]. The positive response after continuous platelet transfusion and concurrent IVIG infusion supports this assumption. We also did not have data on the degree of alloimmunization using methods like the Panel Reactive Antibody assay. This study is limited by its small sample size; however, AITP in the context of allogeneic HSCT is a rare condition, and this case series provides promising evidence to support the use of concurrent IVIG and platelet infusions.

## Conclusions

The findings of this study demonstrate that the administration of continuous IVIG (2 g/kg) along with platelet infusion over 48-hour hours is a safe and effective method that could potentially overcome refractory AITP in allo-HSCT patients and serve as a temporary measure until platelet engraftment can occur. Furthermore, this combined treatment may also be helpful in cases of life-threatening hemorrhages or invasive procedures with a high risk of bleeding.

## Disclosure of prior presentation/publication

This manuscript has not been previously published and has not been submitted for publication elsewhere while under consideration.

## Financial disclosure

This paper did not receive any financial support.

## Ethics committee and institutional review board approval

Ethics approval was obtained by the University of Kansas Medical Center institutional review board.

## Patient consent statement

Subjects gave their informed consent.

## Permission to reproduce material from other sources

Not applicable.

## Clinical trial registration

Not applicable.

## Conflicts of interest

A list of disclosures will be submitted.
